# Rapid Proliferation of Pandemic Research: Implications for Dual-Use Risks

**DOI:** 10.1128/mBio.01864-21

**Published:** 2021-10-19

**Authors:** Sriharshita Musunuri, Jonas B. Sandbrink, Joshua Teperowski Monrad, Megan J. Palmer, Gregory D. Koblentz

**Affiliations:** a Department of Chemistry, Stanford University, Stanford, California, USA; b Future of Humanity Institute, University of Oxford, Oxford, United Kingdom; c Medical Sciences Division, University of Oxford, Oxford, United Kingdom; d Faculty of Public Health and Policy, London School of Hygiene and Tropical Medicine, London, United Kingdom; e Department of Health Policy, London School of Economics, London, United Kingdom; f Department of Bioengineering, Stanford University, Stanford, California, USA; g Center for International Security and Cooperation (CISAC), Stanford University, Stanford, California, USA; h Schar School of Policy and Government, George Mason University, Fairfax, Virginia, USA; Northern Arizona University

**Keywords:** COVID-19, dual-use research, biosecurity, biosafety, pandemic preparedness, preprints, zoonotic risk

## Abstract

The COVID-19 pandemic has demonstrated the world’s vulnerability to biological catastrophe and elicited unprecedented scientific efforts. Some of this work and its derivatives, however, present dual-use risks (i.e., potential harm from misapplication of beneficial research) that have largely gone unaddressed. For instance, gain-of-function studies and reverse genetics protocols may facilitate the engineering of concerning SARS-CoV-2 variants and other pathogens. The risk of accidental or deliberate release of dangerous pathogens may be increased by large-scale collection and characterization of zoonotic viruses undertaken in an effort to understand what enables animal-to-human transmission. These concerns are exacerbated by the rise of preprint publishing that circumvents a late-stage opportunity for dual-use oversight. To prevent the next global health emergency, we must avoid inadvertently increasing the threat of future biological events. This requires a nuanced and proactive approach to dual-use evaluation throughout the research life cycle, including the conception, funding, conduct, and dissemination of research.

## PERSPECTIVE

The COVID-19 pandemic has revealed the world’s vulnerability to biological threats and will shape pandemic preparedness efforts for decades to come. Recent discussions have particularly emphasized biosafety risks associated with gain-of-function experiments and accidental pathogen release (1). However, global health security leaders have also cautioned that the COVID-19 pandemic may increase the threat from deliberate biological events, i.e., biosecurity risks, by potentially inspiring malicious actors (2–4). These warnings come against the backdrop of existing global vulnerabilities to potential biosecurity risks, as both the WHO Joint External Evaluations and the inaugural 2019 Global Health Security Index have identified inadequate capacity and policies for biosecurity in the vast majority of countries (5, 6).

Additionally, biosecurity threats may be particularly concerning given that pathogens engineered for transmissibility or virulence may cause biological events of the largest magnitude, including global catastrophic biological risks (GCBRs) (7). Such engineering may be enabled by the misapplication of publicly available insights and tools from certain “dual-use” life sciences research, even when this research was conceived with beneficial intent. Research associated with the greatest misuse potential constitutes “dual-use research of concern” (DURC), which the U.S. National Institutes of Health defines as “life sciences research that, based on current understanding, can be reasonably anticipated to provide knowledge, information, products or technologies that could be directly misapplied to pose a significant threat with broad potential consequences to public health and safety” (8).

Determining what research exhibits dual-use risks is an ongoing challenge, and if national policies on this exist, they frequently fall short of establishing comprehensive, flexible, and nuanced oversight. In the United States, in addition to the review of risks from public funding of the enhancement of potential pandemic pathogens under the P3CO framework, federally funded institutions are required to assess dual-use risks for only research involving 7 classes of experiments on 15 biological agents, and individual investigators are encouraged to voluntarily raise concerns about research that falls outside these categories (8, 9). Currently, SARS and SARS-CoV-2 are not considered Select Agents under this classification. In contrast, in Canada all institutions working with pathogens and toxins, regardless of funding source, are required to assess dual-use risks of any conducted research (10). According to the Global Health Security Index (GHSI), only 1% of countries worldwide are equipped with adequate review processes for research with especially dangerous pathogens (11). This means that almost all research carried out in the wake of the pandemic will be both conducted and published without adequate dual-use oversight, underscoring the importance of improved guidance globally (Table 1). Moreover, even when review processes are nominally in place, worrying research may nevertheless be conducted in the absence of robust efforts to implement and evaluate the effectiveness of existing policies (12, 13).

**TABLE 1:** Journal articles on SARS-CoV-2 published between 1 January 2020 and 5 July 2021 and GHS Index Dual-Use Indicator by country on a scale of 0 to 100 (low to high preparedness)^*a*^

Country	Research output (no. of articles)	GHS Index Dual-Use Indicator score (0–100)
United States	52,281	50
United Kingdom	21,600	33.3
China	19,389	0
Italy	15,093	0
India	12,896	0
Spain	8,862	0
Canada	8,203	33.3
Germany	7,977	0
France	7,410	0
Australia	7,039	33.3

a Articles were counted if they included “SARS-CoV-2” OR “COVID-19” OR “Coronavirus 2019” OR “novel coronavirus” OR “2019-nCoV,” in title or abstract. The Indicator is determined by whether countries have (a) active oversight of potential dual-use research of concern and (b) screening of genetic synthesis orders against lists of known pathogens and toxins.

The COVID-19 pandemic may exacerbate biological risks stemming from the misapplication of research. We highlight several types of research with dual-use potential associated with pandemic response and preparedness efforts and emphasize how changes to the life science research enterprise complicate oversight of research with dual-use potential. We then describe the need for dual-use frameworks suited for application in the midst of emergency situations, as well as the need to consider dual-use risks associated with pandemic countermeasures. Ongoing dual-use review throughout the research life cycle is necessary to address increasingly common dissemination of research before peer review.

## COVID-19 RESPONSE EFFORTS HAVE CREATED DUAL-USE INSIGHTS

The vast majority of research that is being conducted and published related to the development of countermeasures against SARS-CoV-2 aims to contribute to global pandemic response efforts. This research includes advances such as the identification of neutralizing monoclonal antibodies as therapeutics, genetic surveillance to rapidly characterize variants of concern, and immunogens that aim to elicit lasting protection against the disease (14, 15). However, some work may have dual-use potential that increases the risk of deliberate misuse alongside the potential for accidents, thereby endangering not only the current response but also preparedness efforts for future outbreaks.

For instance, certain research may inform the explicit identification of mutations to the genome of the virus to enhance its resistance to existing countermeasures (16), replicative fitness, or transmissibility. While such studies are often done to pinpoint exactly how current countermeasures, such as convalescent patient sera or monoclonal antibody therapeutics, are insufficient to address potential emerging variants of concern, they also offer a blueprint of changes to be made that could increase the virulence of the virus. Thus, a few of these studies constitute “gain-of-function” (GOF) experiments that have the potential to enhance the lethality and/or transmissibility of a virus. These types of experiments deserve additional review given the associated dual-use risks but may have received less scrutiny due to the urgency of the pandemic and already widespread circulation of the pathogen in question. Some of this information has been rapidly incorporated into countermeasures upon publication and dissemination, such as modification of vaccine formulations to reflect circulating variants (17, 18). However, we must still be wary of the risk that the availability of granular mutational data linked to viral phenotype poses in the long term. This is especially important if it enables the engineering of more concerning strains of SARS-CoV-2, or other viruses, by malicious actors for deliberate release or strategic stockpiling as a biological weapon. While accidental and intentional misuse scenarios may be associated with the same lines of research, the latter could be more catastrophic.

Instructions for the *de novo* reconstruction of replication-competent SARS-CoV-2 virus are another example of dual-use knowledge that has been created and disseminated as a result of the pandemic (19, 20), as they may lower tacit knowledge barriers to conducting risky research. While methods such as restriction enzyme digestion, cDNA fragment assembly, and polymerase chain reaction are staple biochemistry techniques, detailed protocols regarding the assembly of functional virus and its derivative mutants may increase the number of researchers capable of using reverse genetics, regardless of prior training. Therefore, the likelihood that a bad actor acquires the practical knowledge necessary to culture recombinant viruses without safeguards, including those engineered for properties such as immune evasion, increases.

It is important to recognize that transparent dissemination of protocols and reagents is a crucial aspect of accelerating pandemic response research among the scientific community. However, there may be specific tools or insights that pose greater risks than benefits and should require an additional screening step before being shared or should be replaced by a safer alternative. Similar to the practical use of pseudotyped viruses wherever possible to reduce biosafety risks, we should adopt approaches that minimize biosecurity risks. For instance, there are a number of available methods to obtain replication-competent virus other than through synthesis. Extraction of live virus from clinical isolates is not accompanied by straightforward methods of introducing mutations that accentuate certain viral properties in the way that reverse genetics approaches are (21). Another viable alternative may be the use of a transcomplementation system producing nonvirulent SARS-CoV-2 that is infectious for only a single round of replication (22). This approach is also attractive given that it duplicates authentic viral replication, can be implemented in biosafety level 2 (BSL-2) containment, and facilitates the development of countermeasures with fewer risks.

Evidently, only a small fraction of response efforts is associated with dual-use risks. However, we must ensure that such studies do not endanger the overall response and preparedness effort. While an ongoing pandemic warrants rapid dissemination and collaboration to develop countermeasures, maintenance and consideration of dual-use concerns cannot be neglected either in order to avoid the possibility of an even larger crisis in the future.

## COUNTERMEASURE RESEARCH IN THE WAKE OF THE PANDEMIC CAN POSE DUAL-USE RISKS

In addition to the potential dissemination of security-relevant insights during the direct pandemic response, increased infectious disease countermeasure research over the coming years may raise risks from deliberate and accidental biological events. To minimize biosecurity risks from deliberate events, conception and funding decisions regarding infectious disease countermeasure research need to consider how associated insights may inform pathogen engineering by malicious actors. For instance, research on viral vector platform-based vaccines may be associated with generating insights on engineering immune evasion could be translated to pathogens of concern (23). Previous natural exposure to the virus utilized as a vaccine vector may result in preexisting immunity that can limit the effectiveness of vaccination in certain individuals, and induction of antivector immunity through vaccine administration limits the reusability of a given vector platform (24). To overcome this limitation, chimeric vector viruses have been created which evade neutralization by preexisting antibodies (25). While most vaccine-related work focuses on less concerning viral families, such as *Adenoviridae*, researchers have also explored and engineered orthopoxviruses—related to variola virus, the agent that causes smallpox—like vaccinia virus. Less risky alternatives to solving antivector immunity include expanding the vector portfolio to include nonhuman viruses and focusing efforts on nongenetic modifications which are not passed onto viral progeny, such as PEGylation (26, 27). Especially promising may be preferential investment into mRNA-based vaccines which both exhibit excellent properties as fast response platforms and are associated with few dual-use risks (23, 28, 29).

Another example of potentially concerning countermeasure research is the creation of transmissible vaccines for eradicating zoonotic pathogens, which has been advocated for with increased urgency in the wake of the pandemic (30, 31). Despite some potentially useful applications, such research would be associated with substantial safety risks as well as ecological and ethical concerns about introducing a new transmissible agent into animal populations. Importantly, such research would also create unique incentives for engineering the transmissibility, genetic stability, and immune evasion of viruses and hence be associated with significant dual-use risks (32).

## SAFETY AND SECURITY RISKS FROM EFFORTS TO UNDERSTAND ZOONOTIC SPILLOVER EVENTS

Beyond specific countermeasure research leading to dual-use insights on viral engineering, research conducted to investigate and predict zoonotic spillover events may also increase biosafety and biosecurity risks. Experiments that use a “gain-of-function” approach to determine the contribution of genotypic changes to the transmissibility or virulence of a virus could create enhanced potential pandemic pathogens (33), such as the controversial generation of mammalian transmissible H5N1 avian influenza virus (34, 35) as well as more recent work on coronaviruses (36). While this type of research should be conducted at facilities with the appropriate level of safety and security measures, even high-containment labs have an appreciable accident rate (33, 37). Moreover, making specific insights on concerning mutations publicly available can pose information hazards if this enables malicious actors to reconstruct or enhance pandemic pathogens (38, 39).

Systematic approaches to the characterization of viruses with potential for zoonotic spillover bear particular biosecurity risks. Large-scale efforts with the aim to collect hundreds of thousands of samples of viruses and investigate them in laboratories have been proposed and initiated (40). Such efforts are associated not only with accidental exposure and release risks (41) but also the potential of generating dual-use insights. Large-scale characterization of animal viruses may enable computational viral engineering capabilities by creating large data sets which link genetic sequence and function for thousands of viruses. This may be leveraged to create more transmissible and virulent pathogens (42). In addition, broad genomic surveys and characterization of animal viruses have been suggested to be of little practical use to mitigate the emergence of biological events (43). Therefore, preferential investment into approaches which are associated with little biosecurity risk may more robustly reduce overall health security risk. For instance, the real-time surveillance of human populations for emerging pathogens does not involve large-scale collection and characterization of zoonotic viruses and has been highlighted as an effective approach to mitigating outbreaks (44).

Transmissible vaccine research, specific GOF experiments, and large-scale efforts to characterize animal viruses are examples of research aimed at reducing zoonotic risks that at the same time may increase the biological risk from other sources, including deliberate and accidental release. Table 2 summarizes the potential dual-use nature of research across pandemic response and preparedness efforts. Assessing pandemic preparedness research for associated risks should be of particular importance during the coming years, given increased funding for necessary efforts to prevent future pandemics as well as potentially heightened interest in weaponizing viruses by malicious actors, inspired by the havoc caused by the COVID-19 pandemic.

**TABLE 2:** Pandemic response and preparedness research related to SARS-CoV-2 associated with dual-use potential

Work	Proposed benefits	Potential risks	Reference(s)
Identification of mutations that make SARS-CoV-2 more transmissible, virulent, and immune evasive	Informing genomic surveillance and countermeasure design such as vaccines or monoclonal antibodies	May enable engineering of more concerning variants of SARS-CoV-2 or other viruses	Starr et al. (2021) (16)
Publication of detailed SARS-CoV-2 engineering protocols	Increased access to recombinant SARS-CoV-2 for response research	May inform malicious or careless actors on how to create SARS-CoV-2 variants	Xie et al. (2021) (20)
Engineering immune evasion for viral vectors	Improve effectiveness and reusability of viral vector vaccines	Can create transferable insights on engineering immune evasion for pathogens	Sandbrink and Koblentz (2021) (23), Roberts et al. (2006) (25)
Creation of transmissible vaccines	Use for vaccination of animal reservoirs for eradication of zoonotic viruses at risk of spillover	Safety risks; ethical and ecological concerns; may create insights on engineering transmissibility, genetic stability, and immune evasion	Nuismer et al. (2018) (31), Nuismer and Bull (2020) (30), Sandbrink et al. (2021) (32)
Increased gain-of-function work on future potential pandemic pathogens, not limited to coronaviruses	Prediction of zoonotic epidemics, possibility to inform biosurveillance targeting, and design of countermeasures	Risk of accidental exposure and lab release of engineered pathogens; risk of informing the creation of pathogens with enhanced lethality and transmissibility	Herfst et al. (2012) (34), Imai et al. (2012) (35), Casadevall and Imperiale (2014) (74)
Large-scale viral collection and characterization	Prediction of zoonotic epidemics, possibility to inform biosurveillance targeting, and design of countermeasures	Risk of accidental exposure and release; risk of informing viral engineering by creating large-scale data sets connecting sequence and function	Carroll et al. (2018) (40), Monrad and Katz (2020) (41), Carlson et al. (2021) (42)

## A CHANGING LANDSCAPE FOR DURC REVIEW

Changes to how scientific information is disseminated also pose new challenges for managing dual-use risks. From the rapid sharing of the SARS-CoV-2 genome by Chinese researchers (45) to the internationally coordinated vaccine development process, the swift dissemination of knowledge has been a cornerstone of the groundbreaking scientific advances since the beginning of the pandemic. Although this spread of information has been vital for efforts to curtail global outbreaks, the emergency conditions of the pandemic pose distinct challenges from the perspective of managing any emerging dual-use research of concern.

Though dual-use concerns are ideally identified earlier in the research life cycle, in practice many concerns arise or are made apparent when insights are codified for wider release via publication. Only a minority of life science research journals have written policies for assessing dual-use risks (46–48), but the role of journal review has featured prominently in historical controversies over DURC. In cases involving the reconstruction of the 1918 pandemic influenza virus (49), GOF research on avian influenza A/H5N1 (50–52) and A/H7N1 (53, 54) viruses, and the synthesis of horsepox virus (39, 55, 56), editors, journal DURC committees, and external bodies such as the U.S. National Science Advisory Board for Biosecurity (NSABB) ultimately decided in favor of publication of the manuscripts in question. In contrast, the *Journal of Infectious Diseases* decided in 2014 to redact information on key gene sequences from two manuscripts on the molecular characterization of a novel Clostridium botulinum toxin, following consultation between editors, authors, and various U.S. government agencies (57, 58), while another journal previously rejected manuscripts on smallpox and anthrax out of security concerns (59). Irrespective of each specific outcome, the discussions around these cases have emphasized the role of journal review in biosecurity. However, recent developments in publication practices as well as the unique circumstances of public health emergencies pose distinct challenges for this approach to managing dual-use risks.

One such challenge relates to the use of preprint servers such as bioRxiv, medRxiv, and SSRN, which has been steadily increasing in recent years and surged as the COVID-19 pandemic unfolded. Clearly, preprint publishing provides many benefits, including the rapid dissemination, evaluation, and discussion of academic work; open-access research; the facilitation of interdisciplinary collaborations; and benefits for early-career researchers (60–62). However, the discussion around preprints has primarily focused on scientific integrity (63), and scant attention has been given to the implications of preprint publishing for research with dual-use potential (64). While the effectiveness of any peer review, with or without guidance, to reliably identify and resolve dual-use risks remains uncertain, preprint publishing removes a safeguard against the dissemination of potential biosecurity information hazards that cannot be redacted once published on public servers. Therefore, scientists who choose to publish research with dual-use implications must assume a greater responsibility for reviewing the benefits and risks of their work before publication, including consulting with appropriate experts and authorities, and take measures as relevant to minimize the information hazards posed by their research.

Even when manuscripts are not posted to preprint servers, a public health emergency could influence the extent of scrutiny for dual-use risks, either due to accelerated review (65) or because the presence of a significant health threat—rather than a hypothetical or minor one—leads to a higher tolerance for potential risks than under usual circumstances. Consequently, it is critical that scientific journals and external committees are equipped to evaluate dual-use considerations swiftly and in a way that considers how the risks posed by some information hazards may persist longer than any given public health emergency (38).

## THE PATH FORWARD

To safeguard global pandemic response and preparedness efforts, we need to proactively address dual-use risks. Certain elements of a pandemic response, such as the publication of detailed protocols or insights on immune evasion engineering, bear dual-use potential and may increase the risk from deliberate biological events for the foreseeable future alongside accidents in the near term. Therefore, despite the importance of a fast pandemic response, scientists, funders, and publishers should not blindly conduct or publish any and all research that might help with these efforts but still pause and examine individual approaches for risks and benefits. Importantly, deliberative frameworks must be established and incorporated in the life science research cycle now, so as to avoid becoming an unwelcome burden during the next public health emergency and as the life science enterprise grows. Moreover, steps must be taken to ensure that established guidance has the intended effects on shaping scientific efforts. Specifically, it is vital that implementation of the guidelines is continually evaluated in terms of whether the assumptions embedded in their design hold true in practice, including whether they are correctly interpreted and adhered to by laboratory scientists and where ambiguities arise. Realizing the full potential of dual-use policies requires a strong feedback loop between implementation, evaluation, and review (12, 13).

Pandemic preparedness efforts directed at mitigating risks from different sources of biological risks may interfere with each other (66). For instance, large-scale collection of viruses, GOF experiments, and research into acquisition of human transmissibility that is conducted to assess the risk of zoonotic spillovers may increase the pandemic risk from accidental or deliberate releases (67). Consequences of actions by individuals in this space may have global repercussions, necessitating a global dialogue on how to manage tradeoffs from different lines of preparedness. Key drivers of such a global dialogue should be international organizations and scientific bodies including the World Health Organization, the Biological Weapons Convention, and the InterAcademy Partnership. Moreover, commercial, philanthropic, and public funders will need to play a more active role in incentivizing researchers to consider dual-use tradeoffs. To withstand the test of time and future emergencies, such evaluation must consider dual-use risks beyond lists of specific pathogens and existing technologies (68).

The changing landscape for how scientific information is disseminated necessitates a modern approach to managing dual-use research. The growing role of preprint publishing accentuates the disadvantage of relying exclusively on the academic review stage as a filter for biosecurity risks and the importance of evaluating research early on and throughout its life cycle (56, 69). Enabling stakeholders to manage dual-use concerns in a rapidly evolving landscape will require strategies and incentives to increase transparency, information sharing, and education about risk management (70). At the same time, scientific journals continue to have a critical role in shaping norms and incentives in the life sciences, as research typically receives considerably more attention once it is published in prestigious outlets. Consequently, more publishers should follow the example of pioneering journals in the field that already have robust policies for dual-use review (68). Successful efforts from academic journals will also influence the norms governing preprint servers, which could advance innovative practices. At the minimum, these may include providing guidance and conditions for submission of manuscripts including attesting to and disclosing reviews and moving toward implementing screening for biosecurity and biosafety risks in submitted manuscripts where needed. Given that a few prominent servers host the majority of life science preprints, such screening may be a high-leverage avenue for identifying and mitigating potentially concerning research.

Adequately addressing dual-use risks will require updating assessment frameworks, strengthening oversight of life science research from proposal to publication, educating scientists and other stakeholders who shape the scientific landscape about the importance of this topic, and further developing a culture of responsible science (71). The biosecurity community should also recognize that dual-use oversight is not just a scientific and technical matter but also has political and social dimensions, which must be taken into account when designing processes and systems designed to address dual-use concerns (72). Many of the assumptions underlying the effectiveness of our governance strategies for risk management remain untested, and despite calls for applied biosafety and biosecurity research, this work has received little support (9). In particular the social sciences can make an important contribution to designing institutions necessary to monitor, evaluate, and learn from dual-use governance measures (11). Moreover, oversight is only part of what must be a more comprehensive approach that addresses incentives for proactive risk management—including rewarding innovations and highlighting best practices and champions (73). COVID-19 continues to demonstrate the grave costs of pandemic events and that we cannot afford to wait to address dual-use risks until an inevitable, avoidable disaster strikes. The aftermath of this pandemic is an opportunity to proactively increase preparedness for a wide range of potential global catastrophic biological risks.
